# Ethnobotanical and Phytochemical Profiling of Medicinal Plants from Burkina Faso Used to Increase Physical Performance

**DOI:** 10.3390/medicines9020010

**Published:** 2022-01-28

**Authors:** Hemayoro Sama, Modeste Traoré, Samson Guenné, Ibrahim Séré, Adama Hilou, Mamoudou H. Dicko

**Affiliations:** 1 Laboratory of Biochemistry and Chemistry Applied (LABIOCA), University Joseph KI-ZERBO, Ouagadougou 03 BP 7021, Burkina Faso; desmotraore@gmail.com (M.T.); guesams@gmail.com (S.G.); hiloudio@gmail.com (A.H.); 2 Laboratory of Biochemistry, Biotechnology, Food Technology and Nutrition (LABIOTAN), Department of Biochemistry and Microbiology, University Joseph KI-ZERBO, Ouagadougou 09 BP 848, Burkina Faso; 3 General Management of the National Sports Medicine Center, Ouagadougou 03 BP 7035, Burkina Faso; ibrahimsere72@yahoo.fr

**Keywords:** physical activities, exercise, sport ergogenic natural products, doping

## Abstract

**Background:** Some ergogenic medicinal plants are used in exercise and sport in Africa in order to increase sport performance. However, data on their composition and their possible impacts on health are limited. This study was initiated to provide ethnobotanical data on plants traditionally used to optimize physical performance and to perform a qualitative characterization of their main chemical groups. **Methods:** Ethnobotanical surveys in two communes (Dedougou and Nouna), of the region of *Boucle du Mouhoun*, Burkina Faso and phytochemical analyses of the most interesting plants were conducted. **Results:** A total of 50 respondents including traditional hunters *dozo*, farmers, healers, herbalists, marabouts, etc., were interviewed. Fifty-two species used in the optimization of exercise and sports have been identified. The most cited species were *Cassia sieberiana*, *Tamarindus indica*, *Annona senegalensis*, *Gardenia sokotensis*, *Securidaca longepedunculata*, and *Ficus tonningii*. These plants are known to prevent muscle and skeletal disorders, aches and pains, and mental disorders. The study identified several types of plants including those displaying stimulation, anxiolytic, sedative, adaptogenic, or erythropoietic activities. Phytochemical screening revealed the presence of phenolic compounds, alkaloids, terpenes, and steroids, which are similar molecules families of those of doping molecules. Additionally, TLC screening allowed the characterization of numerous terpene and flavonoid compounds including rutin. **Conclusions:** The possible structural similarity of the characterized chemical groups of these species with those of doping families raise concerns about the consequences of their consumption. However, the identification of the active molecules of these species remains to be performed in order to predict the real risks associated with their consumption.

## 1. Introduction

Physical and sport activities (PSA) are inherent parts of human nature. They have a fundamental social role [1,2]. To feed, one must hunt, fish, cultivate, or gather. Leisure activities, traditional games, ritual ceremonies, and other socio-political events are predominantly marked by physical activities such as dance, mask, and sport competitions. PSA are generally considered to be beneficial to health [2]. The World Health Organization (WHO) recommends regular sport practice throughout life [3]. In practicing these activities, practitioners draw on their minds to overcome their physical limitations. They seek to reduce fatigue, to reduce the impact of the effort on their body, and to surpass themselves. They often resort to the use of certain substances to surpass their actual performance. This practice is known as doping.

Doping behavior is a long-standing practice that has been the subject of controversy since the advent of large-scale competitions in the mid-19th century [4]. This practice is based on the use of prohibited substances by athletes in order to improve their physical or mental performance. Doping has developed, diversified, and become wide-spread over time, and, in recent years, has become a public health problem due to its scale and threat to the health of competitors [5]. Indeed, in addition to raising ethical considerations, the use of doping substances exposes the user to numerous health problems such as risk of high blood pressure, pulmonary and cardiac thrombosis, cases of encephalopathy, etc. [5]. These risks result from the toxicity of the products, their lack of purity, the formulations made, the used doses, and the routes of administration [5]. The most commonly used classes of doping molecules are stimulants (amphetamines, cocaine, caffeine, etc.), natural or synthetic narcotics (dextromoramide, diamorphine, etc., and related substances), anabolics, diuretics, peptide and glycoprotein hormones and analogues, etc. [6]. Doping substances, especially androgenic anabolic steroids, can also slow or even stop the growth process in adolescents [5,7]. Other possible adverse effects of the use substance include hypertension, acne, premature baldness, and premature development in adolescents [5]. Psychologically, the use of substance can lead to high aggression and temper tantrums for no apparent reason [7,8]. This situation has led to the restriction of use of most of these compounds.

Even if the concept of doping seems recent, the use of substances or various processes to improve physical performance is inherent to human history [4,7]. Whether it is for sur-vival, daily activities, the conquest of new territories, or to outperform fellow humans, humans have always tried to improve their performance. As far back as written history, and on all continents, traces of what could be considered doping have been found. In the 6th century before Christ, in Europe, Milon de Critone, who was one of the most famous athletes of antiquity, reported the techniques used by Greek athletes at the first Olympic Games. These recipes consisted of concoctions that were supposed to improve their strength and revitalize their blood and were specific to the discipline they practiced. Thus, jumpers had to consume goat meat, boxers bull meat, and wrestlers pig meat. People in Africa have been using kola nuts in chewing form for thousands of years. It is attributed to have aphrodisiac properties as well as an anti-fatigue effect [9].

In Burkina Faso, leisure activities, traditional games, ritual ceremonies, and other sociopolitical events are mainly marked by physical activities such as dances, mask parties, and sports competitions. In the practice of these activities, many plant-based recipes are used to optimize performance. Preliminary studies in about 25 African member countries of the Conference of Ministers of Youth and Sports of the Francophonie [10] were conducted in 2006 and 2007 on the use of herbal recipes and other substances. Results indicated that 6% and 29% of sports competitors used one or more preparations or products derived from plants, respectively, to improve their sports performance and that 17% of the suppliers were traditional therapists [10]. Unfortunately, the composition of these recipes used in the optimization of physical and sports activities and the nature of their bioactive compounds are very poorly documented. Knowledge of the used recipes and the structure of their potentially active molecules would be of capital importance in assessing the health risks associated with their consumption. The present study was initiated to provide ethnobotanical data on plants traditionally used in Burkina-Faso to optimize physical activities and to carry out the characterization of the main chemical groups they contain. That will allow establishing a structural comparison between these groups and knowing doping molecule families in order to assess the health risks associated with their consumption by practitioners.

## 2. Materials and Methods

### 2.1. Study Area

**Figure 1:**
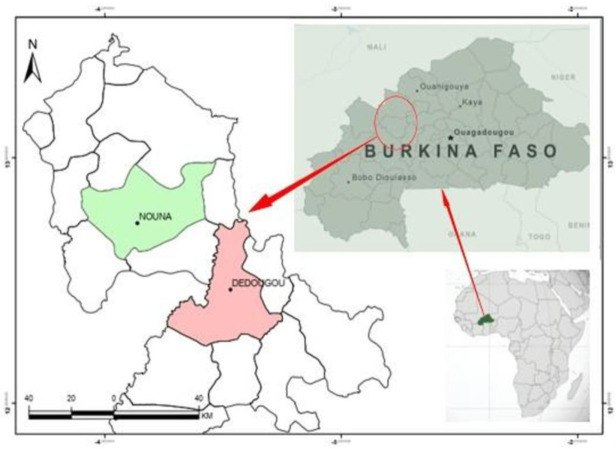
Location of the communes of Nouna and Dedougou.

The study was conducted in two communes (Figure 1) of the region of *Boucle du Mouhoun*, Burkina Faso: Dedougou and Nouna. The commune of Dedougou covers an ar-ea of 1352.56 km^2^. It is located at 230 km from Ouagadougou and 175 km from Bobo Dioulasso. Its population is estimated at 86,965 inhabitants in 2006 and knows an important ethnic and socio-cultural mix, mainly made up of Bwaba (31.80%), Mossi (26.91%), Marka (12.08%) and Samo (10.70%). Its inhabitants represent 6% of the population of the region of *Boucle du Mouhoun*. The commune of Nouna has a population of 73,006 inhabitants with an area of 1 065 km^2^. It totals 5.1% of the population of the *Boucle du Mouhoun* region. It is mainly populated by the ethnic groups of San, Marka, Fulani, Bwaba, Bobos, and Mossi.

### 2.2. Data Collection

The ethnobotanical survey was run between September 2019 and August 2020. Prior contact was made with resource persons (village advisors, village chief, and village council leaders) in the various villages of the two communes to inform them on the objectives of the study and to identify potential participants (traditional practitioners, herbalists, traditional hunters, and older people). People were then approached on the basis of a dialogue in the language of their choice following a questionnaire (in French language, available in Supplementary Materials). Interpreters were often needed. A field trip was then organized, and the plants mentioned in the interview were identified and collected with the help of the interviewees. The mentioned plant specimens were collected, identified, and deposited in the herbarium of the Laboratory of Plant Biology and Ecology of the Joseph KI-ZERBO University, Burkina Faso.

### 2.3. Phytochemical Analysis

The phytochemical screening was carried out on the basis of citation frequencies and the literature search. Indeed, after the bibliographic research [11,12,13], *Tamarindus indica*, which has a high citation frequency, was discarded because the species has already been the subject of many studies. Thus, six of the most interesting species, including *Annona senegalensis* Pers, *Bauhinia thonningii* (Schumach.) Milne-Redh., *Cassia sieberiana* DC, *Ficus thonningii* (Blume), *Gardenia sokotensis* Hutch, and *Securidaca longepedunculata* Fres were selected for the phytochemistry analyses. The leaves of *Ficus thonningii* were collected in November 2020 in the village of Mani (Latitude: 12°74′33″ North; Longitude 3°87′84″ West) in the commune of Nouna while leaves of *Annona senegalensis* Pers, *Bauhinia thonningii* (Schumach.) Milne-Redh, *Cassia sieberiana* DC, *Gardenia sokotensis* Hutch, and *Securidaca longepedunculata* Fres were collected in the same period in the village of Yonkuy (Latitude: 12°6008 North; Longitude: 3°47′ 3789) in the commune of Dedougou. The collected material was placed in plastic bags and transported to the laboratory to be dried and crushed.

#### Extractions

Two types of extraction were made. For maceration, 25 g of plant powder of each species was homogenized in 250 mL of 50% ethanol under mechanical stirring for 24 h. The filtrate was centrifuged, the solvent was evaporated with a rotavapor, and the extract was placed in the oven at 40 °C until drying. For decoction, 25 g of plant powder of each species (leaves) is put in a flask in which 250 mL of water was introduced and then placed on a hot plate. After 15 min of boiling, the decoction was left to cool to room temperature and then filtered, and the filtrate was centrifuged and placed in the oven at 50 °C for drying. Extraction yield was calculated using the following formula:

Yield = (Mass of dried residue ∗ 100)/Mass of plant powder

### 2.4. Phytochemical Screening

Phytochemical screening aimed to characterize the main chemical groups of bioactive compounds present in the different plant extracts. For our study, this screening is carried out from the 50% hydroalcoholic macerates and the decoctions. For this purpose, 100 mg of each residue was dissolved in 10 mL of solvent (50% hydroalcoholic for macerate extracts and distilled water for decoction) to serve as a stock solution for the phytochemical screening. Qualitative tests were carried out according to methods de-scribed in the literature [14,15].

To characterize flavonoids by the Shibata test, 1 mL of each extract was introduced into a test tube with 1 mL of distilled water. Fragments of magnesium turnings and four drops of HCl (concentrate) were added to each tube. After 10 min, the appearance of a red color indicated the presence of flavones, the appearance of a purple color indicated the presence of flavonols, and the appearance of a purplish red color indicated the presence of flavanones and flavanols [15].

To detect tannins and polyphenols by the FeCl_3_ test, 1 mL of each extract was intro-duced into a test tube with 1 mL of distilled water; then, two to three drops of 1% FeCl_3_ were added. The appearance of a blue-black or green-blackish coloration indicates the presence of gallic tannins or ellagic tannins, respectively [15].

Saponosides were detected by the foam test. An extract/distilled water mixture (1/1 *v*/*v*) was introduced into a test tube. The tube was then shaken vigorously. The appearance of a column of foam at least 1 cm high, persisting for at least 15 min indicates the presence of saponosides [15].

Dragendorff test was used to characterize alkaloids. To detect alkaloids, 1 mL was introduced in a test tube with 1 mL of hydrochloric acid then heated in a water bath. The obtained solution is divided in two test tubes with equal volume. The first tube is used as a control and in the second one Dragendorff’s reagent (potassium tetra iodo bismutate) is added. The appearance of a yellowish-white precipitate shows the presence of alkaloids [16].

Triterpenes and steroids were detected by Libermann–Burchard test. This test consists in introducing 1 mL of each extract in a test tube with a mixture (1 mL) acetic anhy-dride/chloroform (1:1 *v*/*v*), then 1 mL of concentrated sulfuric acid (H_2_SO_4_) is delicately added in the tube. The presence of sterols and triterpenes in the extracts is indicated by the appearance of a red-brown ring at the interface zone of the two liquids.

### 2.5. TLC Screening

TLC screening was carried out to detect flavonoids, triterpenes, and steroids as a confirmation of the tube tests. TLC 10 cm × 10 cm silica gel plates were used for TLC. For each sample, the deposition was performed manually using capillary tubes. After deposition, the plates were placed in TLC tanks. After saturation, the plates were air dried and then sprayed with Neu’s reagent for flavonoids (Table 1). For steroids and triterpenes, the plates were sprayed with Libermann–Burchard reagent and heated at 100 °C for 10 min. The plates were then visualized at 366 nm under UV.

### 2.6. Data Analysis

The survey data was entered into Excel to calculate the frequency of citation of the different species. The frequency of plants citation was calculated according to the formula:

F = (Number of respondents citing the species ∗ 100)/Total number of persons interviewed

The data were also used to calculate the ethnobotanical value use (VU) of the different species. Determining the use value of different species allows to assess the importance of the species for the relevant use category to be assessed. The use values of the different species were calculated according to the formula:

VU = (Use score given by respondents)/Total number of persons interviewed

## 3. Results

### 3.1. Ethnobotanical Survey Data

#### 3.1.1. Socio-Demographic Profile of Survey Respondents and Physical and Sporting Activities Practiced in the Region

A total of 50 respondents were interviewed, 88% of whom were men and 18% women. The respondents were traditional hunters “Dozo” (42%), farmers (24%), healers (16%), herbalists (14%), marabouts (2%), and fractures reducers (2%). Their ages ranged from 21 to 103 years. Their average age was 60 years. The origin of knowledge was based on inherited knowledge (46%), inheritance and training (34%), and training (20%). The respondents are divided into seven ethnic groups, namely Marka (36%), Mossi (18%), San (16%), Bwaba (16%), Peulh (8%), Bobo (4%), and Koo (2%). Among respondents, 76% were illiterate. The survey revealed that various physical and sporting activities were practiced in the two communes of the region of “Boucle du Mouhoun”. The main traditional physical activities practiced in the two communes are traditional dances, agricultural activities, mask making, and hunting. The sports activities practiced in the localities are football and cycling.

#### 3.1.2. Pathologies and Therapeutic Effects and Preparations Identified

The survey also revealed that various herbal preparations were traditionally used in the practice of physical activities. These preparations were used against the main health problems and to optimize performance. The main health problems encountered in the practice of physical activities and sports are fractures, strains, wounds, aches, and psychological disorders (stress and anxiety). These preparations are, therefore, used against aches and pains and fatigue and to increase muscle strength and endurance in the production of physical effort. The therapeutic effects sought in the various preparations of plants are multiple and closely related to these health disorders.

#### 3.1.3. Identified Species in the Survey

A total of 52 plant species were identified during the survey (Table 2). These plant species are distributed in twenty-six families. The most represented families are Anacardiaceae (five species), Combretaceae (six species), Caesalpiniaceae (six species), and Mimosaceae (five species). The most cited species are *Tamarindus indica* L. (22%), *Cassia sieberiana* DC (22%), *Gardenia sokotensis* Hutch (18%), *Ficus tonningii* Blume (18%), *Gardenia sokotensis* Hutch. (15%), *Annona senegalensis* Pers. (16%), *Securidaca longepedunculata* Fres (16%), *Bauhinia tonningii* (Schumach.) Milne-Redh. (16%), and *Ficus thonningii* Miq. (14%). All parts are used either alone or in association with one or more other parts. The most used parts of the plants are roots (70%), leaves (46%), and barks (26%), while the least used parts are root barks, leaves, and barks and the whole plant, with a frequency of 1%. Ten modes of administration are cited. The most cited modes of administration are bathing and drinking (45%), oral administration (20.40%), and application (7%), while the least cited are chewing and sucking, with a frequency of 1%.

### 3.2. Results of Phytochemical Analysis

**Figure 2:**
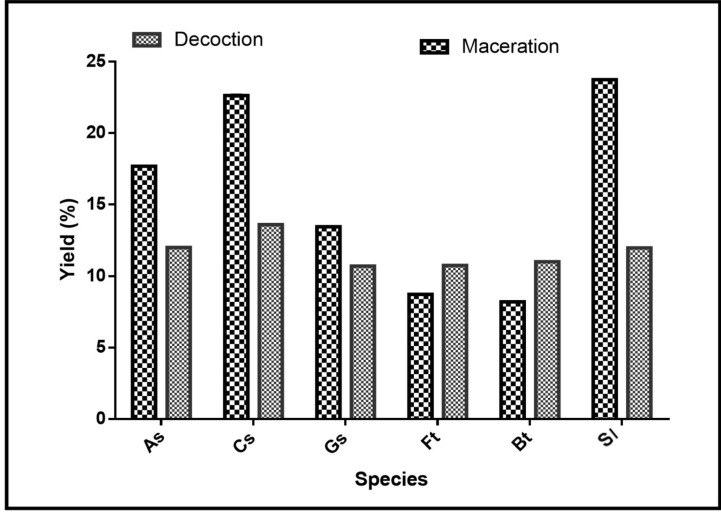
Extraction yields by species. As: *Annona senegalensis*; Cs: *Cassia sieberiana*; Gs: *Gardenia sokotensis*; Ft: *Ficus tonningii*; Bt: *Bauhinia tonningii*; and Sl: *Securidaca longepeculata*.

To characterize chemical active compounds, extractions were made. Figure 2 shows the extraction yields of the different extracts. The ethanolic macerates of *Securidaca longepecudulata* (23.73%), followed, respectively, by *Cassia sieberiana* (22.64%), *Annona senegalensis* (17.68%), and *Gardenia sokotensis* (13.45%), presented the best yields. For *Ficus thonningii* and *Bauhinia thonningii*, the highest yields were obtained with the decoctions with, respectively, 10.75% and 11% yields against 8.72% and 8.2% for the lowest.

The phytochemical screening (Table 3) indicated the presence of tannins, flavonoids, coumarin, and steroids and terpenes in all extracts. Alkaloids were detected only in macerates of *Annona senegalensis*, *Cassia sieberiana*, and *Ficus thonningii* and in decoction of *Gardenia sokotensis* and *Securidaca* *longepedunculata*. Saponosides were only not detected in the two extracts of *Ficus thonningii* and the decoctate of *Bauhinia thonningii*.

**Figure 3:**
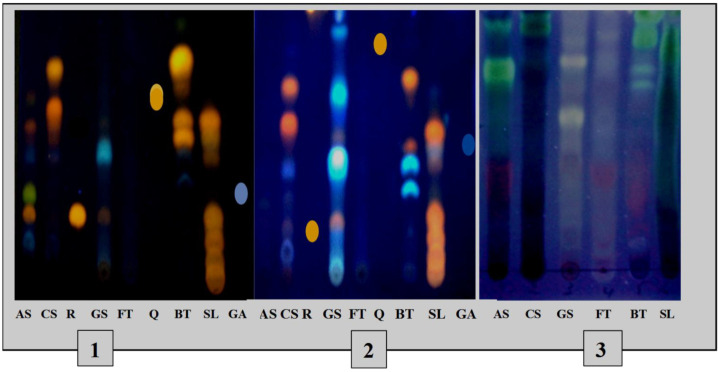
TLC profiles of phenolic and terpene compounds of the species studied. 1: TLC profile of phenolics from decocted extracts; 2: TLC profile of phenolics from macerated extracts; 3: chromatogram of triterpenes and steroids. As: *A. senegalensis*; Cs: *C. sieberiana*; R: rutin, Gs: *G. sokotensis*; F.t: *F. thonningii*, Q: quercetin, B.t: *B. thonningii*, S.l: *S. longepedunculata*, GA: gallic acid.

The TLC profiles (Figure 3) of the phenolic and terpene compounds vary according to the species and the extraction mode. The chromatogram shows the presence of different phenolic compounds in the different extracts with, notably, the presence of rutin in the decocts of *Annona senegalensis* and *Securidaca* *longepedunculata* and *Gardenia sokotensis* and quercetin in the macerated *Bauhinia thonningii*. The analysis of terpene compounds also allowed the characterization of some terpene compounds in various plant species including *Annona senegalensis*, *Securidaca* *longepedunculata*, and *Bauhinia thonningii*.

## 4. Discussion

This study aimed to identify plants traditionally used in the optimization of physical and athletic activities and to qualitatively characterize the main chemical groups they contain. Respondents participated freely to the study. The refusal rate was 12%. The predominance of men (88%) observed in our survey population could be explained by the culture in these societies, where daily physical activities are mostly dedicated to men, women being rather busy with household tasks. Similar results were reported by [17], whose work showed that, in most African societies, physical activities are generally dedicated to men. The study reported that knowledge of traditional pharmacopoeia comes from inheritance. Furthermore, the study reported that physical activities were practiced as part of routine (e.g., farming, cultural, and leisure) activities. Traditional and cultural activities are the most important, although there are some modern sports activities including football and cycling. This reflects the importance of filial transmission of traditional and cultural knowledge and practices in African society [17].

The study revealed that the practice of physical and sports activities is generally associated with disorders including musculoskeletal disorders (fractures, strains, and aches) and psychological disorders (fear, stress, and anxiety) but also the desire to optimize performance by the consumption of certain plants. To deal with these disorders, practitioners use recipes, generally based on plants. Fifty-two (52) plant species were thus identified, and the most cited species were *Tamarindus indica* L. (22%), *Cassia sieberiana* DC (22%), *Gardenia sokotensis* Hutch (18%), *Ficus tonningii* Blume (18%), *Gardenia sokotensis* Hutch. (15%), *Annona senegalensis* Pers. (16%), *Securidaca longepedunculata* Fres (16%), *Bauhinia tonningii* (Schumach.) Milne-Redh. (16%), and *Ficus thonningii* Miq. (14%). The use of various recipes by sports practitioners, especially athletes, goes back a long way. These recipes were supposed to improve their strength and renew their blood and were specific to the discipline practiced. Although data on the use of plants in the optimization of physical and sports activities is very limited, studies have reported the use of various plant species not directly in the practice of these activities but in the management of numerous disorders, some of which are associated with the practice and optimization of physical and sports activities. These include musculoskeletal disorders, psychological disorders, stimulation of physical activity, and anti-fatigue effects. Thus, some plant species are traditionally used against muscular disorders [18], musculoskeletal, skin, sensory, nervous, and respiratory disorders and wounds [19,20], aches and pains, asthenia [21], pain, myalgia and rheumatism [22,23], and heart pain [23,24]. The different species reported in the study are divided into different categories of plants according to their biological properties.

**Figure 4:**
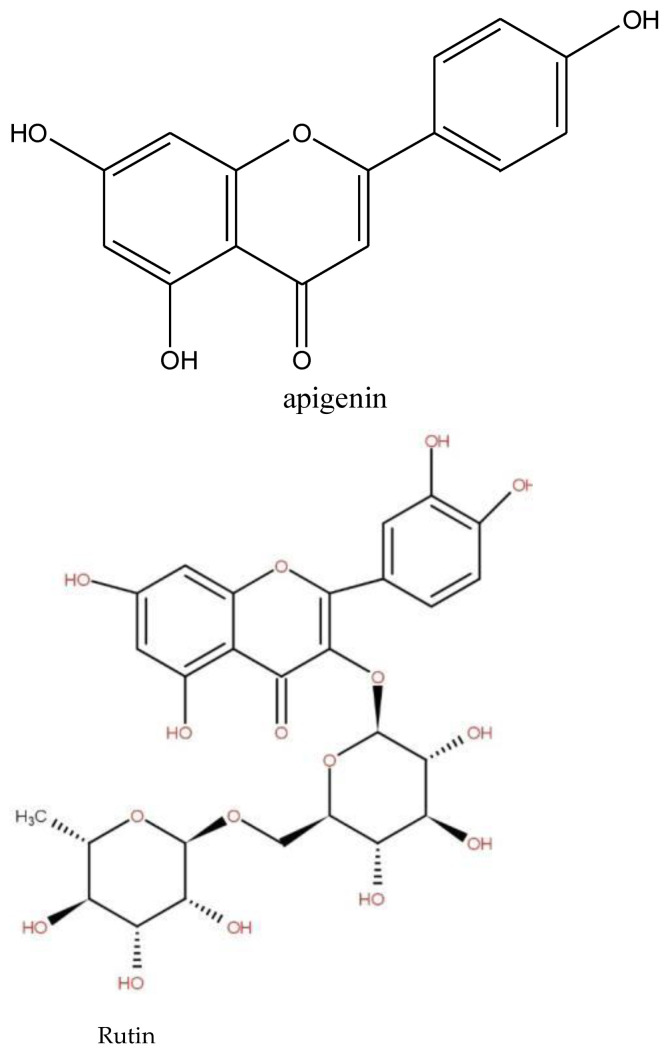
Structure of some stimulating, anxiolytic, sedative, and anticonvulsant properties isolate in medicinal plant. The second type was plants with adaptogen properties.

The first type of identified plants was those rich in compounds, which display stimulating, anxiolytic, sedative, and anticonvulsant properties [25,26,27]. Herbs with these properties combat anxiety. Many medicinal plants reported by the survey (Table 2) are known to have an anxiolytic effect. The aqueous root extract of *Securidaca longepedunculata* is used in traditional medicine in the management of epilepsies and psychosis [25]. The aqueous root extract of *Securidaca longepedunculata* has shown anticonvulsant, anxiolytic, and sedative actions. The anticonvulsant activity with pronounced hypnotic and muscle relaxant of the methanol root bark extracts of *Annona senegalensis* was reported [27,28]. Species of the genus *Annona* generally consumed as fresh fruits are also widely used in folk medicine. *A. senegalensis* possesses central depressant, anticonvulsant, tranquilizing, and anxiolytic properties. Some species reported by the survey are reputed to also have stimulating and tonic properties for the body [20,22]. The bark of the *Tamarindus indica* tree is regarded as having effective tonic, astringent, and febrifuge properties and is used as a tonic and in lotions or poultices to relieve sores, ulcers, boils, and rashes. The dried pulp of the fruit is used to cure exhaustion, giddiness, mental fatigue, and morbid thirst [26]. *Capparis sepiaria* fruits are taken with water as a tonic [29]. Some species of the genus *Bauhinia* is known for its therapeutic activity and are used as tonic and astringent [29]. These stimulating and anxiolytic properties could therefore be attributed either to their chemical composition. These species contain psychotropic substances such as alkaloids, phenolics, terpenes, and saponins detected in some reported species, with a central nervous system depressant action, designed to reduce or eliminate the symptoms of anxiety, in the hope of not producing sedation or sleep [30]. Previous works reported the sedative and anxiolytic properties due to the affinity of the GABAergic system for the benzodiazepine region of their compounds [31]. The anxiolytic, sedative, and anticonvulsant, properties activities of Annona senegalensis were attributed to flavonoids in the leaves [27,28]. Phenolic compounds, especially flavonoids, display anxiolytic, sedative, anticonvulsant, and analgesic properties by interacting with various receptors and signaling systems, including GABA receptors [32,33]. Previous studies showed that some flavonoids such as rutin (Figure 4) detected in our study in the decocted extract of *Annona senegalensis* and apigenin strongly modulated IGABA (GABA-induced chloride current) [31]. Phenolic and flavonoids were detected in all of the six species concerned by the phytochemical screening. Alkaloids, detected in some reported species such as *Annona senegalensis*, *Cassia sieberiana*, *Ficus tonningii,* and *Bauhinia tonningii*, were reported by [34] to be the main responsible agents of the anticonvulsant and anxiolytic properties. Terpenes detected in all of the six species related to the phytochemical analyses (Table 3) have been reported to have anticonvulsant and anxiolytic activities with pronounced hypnotic and muscle relaxant effects [30].

**Figure 5:**
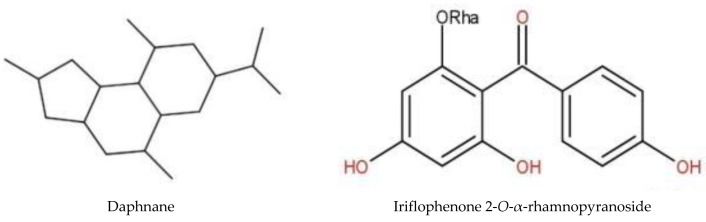
Structure of some adaptogen molecules.

Adaptogen plants are plants that contain bioactive compounds capable of inducing a state of increased non-specific resistance in an organism, allowing it to counteract stress signals and adapt to exceptional effort. An adaptogen enhances an organism’s response to all stresses, whether caused by infectious agents or not, which differentiates it from an immunostimulant. In addition, adaptogens have a broad spectrum of activity [35]. The most common adaptogen plants are *Panax ginseng*, *Eleutherococcus senticosus*, *Rhodiola rosea, Schisandra chinensis* (Turcz.) Baill, *Tribulus terrestris* L., *Astragalus propinquus* Schischkin (syn. A. membranaceus), *Withania somnifera* (L.), *Phaleria nisidai* and *Lepidium meyenii* Walp [35], which are not recorded in this study. In our study, species such as *Cymbopogon giganteus*, *Calotropis procera*, *Cassia sieberiana*, and *Ficus tonningii*, mentioned in the survey, are known for their adaptogen properties. A leaf infusion mixed with plant parts of *Cymbopogon giganteus* Choiv. (Poaceae) is used as a diuretic in edema and for antihypertension. Another source [36] stated that the plant is an astringent and adaptogenic. *Calotropis procera* is known for its hepatoprotective, antioxidant, antipyretic, and anti-inflammatory properties. The anti-stress potential of many species of the genus Ficus, genus of *Ficus tonningii*, has been reported. Additionally, the chemical families of some adaptogen molecules isolated to plants (Figure 5) are terpenes and phenolics. Phenolic and terpenes were detected in *Cassia sieberiana*, *Gardenia sokotensis*, *Ficus tonningii*, *Gardenia sokotensis*, *Annona senegalensis*, *Securidaca longepedunculata*, *Bauhinia tonningii*, and *Ficus thonningii*, Phenolic compounds have antioxidant activities, are inhibitors of ROS-generating enzymes, and induce the biosynthesis of antioxidant enzymes [37]. Phenolic compounds, especially flavonoid-rich adaptogen companions, improve brain function; act as powerful antioxidants; enhance the antioxidant effects of vitamin C; regulate nitric oxide, a potent free radical that helps regulate blood flow; protect heart health by preventing blood clots, protecting against oxidation of LDL (“bad cholesterol”) and lowering high blood pressure; improve sexual functionand also reduce inflammation and bolster immune function [35]. This is the case of some flavonoids used in the treatment of disorders related to capillary fragility [38]. Rutin is also known to have high antioxidant properties. Steroids and triterpenes have hypotensive, analgesic, and free radical scavenging properties [22].

The third type was plants with erythropoetic activity. These plants, such as erythropoietin (EPO), a hormone naturally secreted by our body, stimulate the formation of red blood cells. EPO is mainly used for endurance sports: cycling, athletics, cross-country skiing, etc. It increases the maximum power developed and the maximum oxygen consumption and improves endurance. EPO is on the World Anti-Doping Agency’s list of prohibited substances. Some herbals cited in our study, such as *Ficus thonningii* and *Cassia sieberiana*, are known to stimulate hematites and protect the erythrocyte membrane [39]. These species are known for their anti-anemic and anti-sickle cell properties. Indeed, the hydroethanolic extract of *Cassia sieberiana* stem barks and roots significantly increased red and white blood cells, hemoglobin, and triglycerides [40]. Supplementation of goat alimentation by different concentrations of the hydroethanolic extracts positively influenced the hematology parameters of the animal blood [41]. The leaves and root bark of *Calotropis procera* are used to treat sickle cell disease [22]. *Acacia nilotica* root extracts produced significant increase in red blood cells, white blood cells, hemoglobin (Hb), and packed cell volume compared to the control in male rats, also preventing hypercholesterolemia by reducing serum cholesterol [42]. The serum cholesterol-lowering effect of the aqueous extract may be attributed to the presence of saponins identified as one of the phytochemicals in the root of this plant [41]. In our study, saponins were detected in all the phytochemical studied extracts without the two extracts of *Ficus tonningii* and the decoction of *Bauhinia tonningii*. Plant saponins have been reported to inhibit cholesterol absorption from the intestinal lumen in experimental animals and consequently reduce the concentration of serum cholesterol. This may be due to the ability of saponins to complex with cholesterol in the digestive tract or a direct effect of plant saponins on cholesterol metabolism [41]. Additionally, the fact that steroidal terpenes, the chemical family of EPO, are detected in all the species that were chemically screened suggests that these species may contain this molecule or its analogues [17,43].

The phytochemical screening of the extracts of some reported species choice based on their frequency of citation, use value, and bibliographic research [11,13,26] revealed the presence of bioactive compound groups. The main chemical groups are phenolics, coumarins, alkaloids, triterpenes, and steroids. The main characterized classes in these plants in our study are known for their biological properties [44]. Structurally, the most represented molecules among doping molecules in sport are alkaloids, steroids, and phenolic compounds. Indeed, stimulants such as amphetamine, caffeine, and diuretics such as triamterene are nitrogenous compounds, while glucocorticoids such as cholesterol and cortisone, as well as some compounds such as quinolones and nadrolone, which are anabolic agents, are phenolic-like in nature. Additionally, some doping hormones such as erythropoietin are steroidal or terpenic in nature. The importance of the biochemical potentials of the plants studied could justify the contribution of these plants to the maintenance or re-establishment of normal physiological conditions essential not only to health but also and especially to any physical effort. This would explain their use in optimization of physical activity to combat fatigue and pain and thus improve physical performance. These results reinforce our hypothesis that there would be a structural link between synthetic doping molecules and the active principles of plants used to optimize physical activity. Therefore, further analysis could provide information on the nature (doping or not) of the active ingredients of these compounds and their consequences on consumers.

## 5. Conclusions

The study allowed to provide ethnobotanical data on plants traditionally used to optimize physical activities in order to qualitatively characterize the main chemical groups they contain and establish a structural comparison between their structure and known doping molecule families. The survey carried out in the communes of Dedougou and Nouna, in the region of *Boucle du Mouhoun*, confirmed the use of plants to improve performance in agricultural, cultural, and/or ritual activities and entertainment activities. These plant species are known for their biology properties including stimulating, anxiolytic, sedative, adaptogenic, or erythropoietic activities. On the basis of the frequency of citation, the value of use of the identified species and bibliographic data, the most interesting species were selected and subjected to phytochemical screening. Phytochemical screening revealed the presence of phenolic compounds, alkaloids, terpenes, and steroids, which are families of some doping molecules. The possible structural similarity of the compounds containing chemical groups of these species to those of doping molecule chemical groups raises concerns about the consequences of their consumption. However, further analysis to identify the structures of active constituents of the different species is needed to reach a more exhaustive conclusion on the bioactivities associated with their consumption.

## Figures and Tables

**Table 1:** Systems and revelators used for TLC.

Bioactive Compounds	Revelators Used	Colour Observed Under UV
Flavonoids System: ethyl acetate/formic acid/acetic acid/water (100/11/11/26 *v*/*v*/*v*/v *)	Neu’s reagent	Yellow and yellowish green coloration.
Triterpenes and steroids Chloroform/ethyl acetate/formic acid (10/90/20; *v*/*v*/*v*)	Libermann–Burchrad reagent	Blue violet coloration or Red to blue violet

* v= volume.

**Table 2:** Different species recorded.

Scientific Names (Familly)	Local Names	Parts Used	Method of Preparation	Method of Administration	Associated Plants	Indication	* F(%)	** VU (%)
*Cassia sieberiana* DC (Ceasalpiniaceae)	Sindjan (d)	Bark, root, leaves.	Decoction, Maceration	Bathing, drinking	*Bauhinia thonningii*, *Annona senegalensis*	Ache, anxiolytic, pain	22	34
*Tamarindus indica* L. (Césalpiniaceae)	Tomi(dioula)	Fruit, root, sap	Decoction	Bathing, drinking, incensing		Wounds, anxiolytic, psychotropic	22	32
(Rubiaceae)	Toukorogulen (d); Tang-ra-kweenga (mo)	Fruit, leaves, root, shaft,	Decoction, maceration	Bathing, drinking	*Tamarindus indica*, *A. Erythrocalyx*	Ache, anxiolytic, psychotropic	18	28
*Annona senegalensis* Pers. (Annonaceae)	Mandesunsun(d)	Leaves, root,	Decoction, cullet, maceration, powder,	Bathing, drinking, massage	*Zinziber officinale*	Ache, pain, wounds, psychotropic	16	16
*Securidaca longepedunculata* Fres (Polygalaceae)	Toumouni (b); Joro (d)	Leaves, root,	Decoction, maceration, powder	Bathing, drinking	*Nauclea latifolia*, *Cassia Sieberiana*, *KOH*	Ache, wounds., anxiolytic, pain	16	18
*Bauhinia thonningii* (Schumach.) Milne-Redh. (Ceasalpiniaceae)	Gnama (d)	Leaves, fruit	Decoction	Mastication, drinking		Ache, wounds, strengthening,	16	14
*Ficus thonningii Miq*. (Moraceae)	Kognojidiba (marka)	Bark, leaves, root,	Decoction	Bathing, drinking		Ache, anxiolytic	14	12
*Guiera senegalensis J.F. Gmel.* (Combretaceae)	Kunguè (marka)	Bark, leaves, root, fruit	Decoction	Bathing, drinking	*Kaya senegalensis*, *Tamarindus indica*	Ache, psychotropic	12	20
*Khaya senegalensis (Desr.) A Juss*. (Meliaceae)	Jalan(d)	Latex, bark.	Decoction	Bathing, drinking	*T. indica*, *G. senegalensis*; *milk*	Psychotropic, wound, aches, anxiolytic	10	14
*Ozoroa insignis* Del (Anacardiaceae)	Plakoossi (m)	Leaves, root.	Decoction, P	Bathing, drinking	*Phoenix dactylifera*	Ache, psychotropic	10	12
*Parkia biglobosa (Jacq.) R. Br. ex G. Don* (Mimosaceae)	Néréyiri(d)	Bark, root, fungius, parasite	Decoction., cullet	Sucking, drink,		Psychotropic, anxiolytic, wounds	10	16
*Bauhinia thonningii* (Schumach.) Milne-Redh. (Ceasalpiniaceae)	Gnama (d)	Leaves, fruit	Decoction	Mastication, drinking		Ache, wounds, strengthening	8	14
*Sterculia setigera Del*. (Sterculiaceae)	Koflaba ()	Bark	Decoction	Application, massage		Wounds, foulure	8	8
*Anogeissus leiocarpa (DC.) Guill. & Perr*. (Combretaceae)	Lôo (S), kèkèba (m)	Bark of root	Decoction	Bathing, drinking	*Gardenia sokotensis*	Ache, anxiolytic	6	6
*Capparis sepiaria L*. (Capparaceae)	Trigui (d)	Leaves, sap	Decoction	Bathing, drinking, massage		Ache, psychotropic, sprain	6	10
*Entada africana* Guill. et Perr. (Mimosaceae)	Samaninnin (d)	Bark, root,	Decoction, latex	Bathing, application		Wounds, pain, strengthening	6	12
*Flueggea virosa* (Roxb ex Wllld.) Voigt (Euphorbiaceae)	Bagana	Leaves	Decoction	Drinking	*Tamarindus indica*	Ache	6	6
*Sclerocarya birrea* (A. Rich.) Hochst. (Anacardiaceae)		Bark, mistletoe, root	Decoction, powder	Application, Bathing, drinking		Ache, wounds, psychotropic	6	6
*Trichilia emetica Vahl* (Meliaceae)	Lonlôron (s), Sulafinsan (d)	Bark, leaves, root	Decoction, maceration	Bathing	*Gardenia sokotensis.*	Ache, anxiolytic, pain	6	14
*Acacia erythrocalyx* Brenan (Mimosaceae)	Sansan (d)	Root,	Maceration	Bathing, drinking		Ache	4	4
*Acacia nilotica (L) Willd. ex* Del (Mimosaceae)		Fruit	Decoction	Application		Wounds	4	4
*Bombax costatum* Pellegr. et Vuillet (Bombacaceae)		Bark	Powder	Application		Wounds	4	4
*Calotropis procera* (Ait) Ait. F. (Asclepiadaceae)	Fogofogo (d)	Root.	Cullet	Massage		Ache, wounds	4	8
*Citrus limon IL.)* Burm. F. (Rutaceae)	Lemuru (d)	Leaves, sap	Decoction	Bathing, fumigation		Ache, psychotropic	4	4
*Combretum micranthum* G. Don. (Combretaceae)		Leaves	Decoction	Bathing, drinking		Ache, pain	4	4
*Daniellia oliveri* (Rolfe) Hutch et Dalz (Ceasalpiniaceae)	Aoga(mo)	Bark	Powder	Massage	*Ximenia americana*	Ache, anxiolitic	4	4
*Detarium microcarpum* Guill. et Perr (Ceasalpiniaceae)	Tomokunba (m)	Bark, Leaves, roots	Decoction	Bathing, drinking		Ache; psychotropic	4	6
*Erythrina senegalensis DC*. (Fabaceae)	Tièpereba (marka)	Root.	Decoction	Bathing, drinking		Ache, sprain	4	4
*Icacinia Senegalensis* (Icacinacea)e	Mankana (d)	Tuber	Chewing			Pain, psychotropic	4	4
*Lannea acida* A. Rich. (Anacardiaceae)		Bark	Decoction	Bathing		Wounds	4	4
*Nauclea latifolia* Sm. (Rubiaceae)			Decoction	Bathing, drinking		Ache, anxiolytic	4	6
*Stereospermum kunthianum* Cham. (Bignoniaceae)	Ninyilenga (Moore),	Leaves.	Decoction	Drinking, bathing		Strengthening,	4	6
*Ximenia americana* L. (Olacaceae)		Root, sap	Decoction, powder	Massage, bathing, drinking		Ache, psychotropic	4	4
*Acacia seyal* Del. (Mimosaceae)	Wanigwe (d)	Sap	Powder	Encensement		Psychotropic	2	2
*Balanites aegyptiaca (L) Del.* (Balanitaceae)	Seguinnin (d)	Parasite	Maceration, powder	Drinking		Psychotropic	2	4
*Cassia alata L.* (Caesalpiniaceae)		Leaves	Decoction	Bathing, drinking		Psychotropic	2	2
*Cochlospermum planchonii* Hook f. ex Planck (Cochlospermaceae)	N’Dribla	Root	Powder	Application	*Cassia sieberiana.*	Wounds	2	2
*Combretum nigricans* Lepr. Ex Guill. Et Perr. (Combretaceae)	Toupin(m)	Leaves	Decoction	Bathing	*Trichilia emetica*	Wounds	2	2
*Cymbopogon giganteus* Chiov. (Poaceae)	Thienkala (d)	Whole plant	Fresh plant	Application	*Euphorbia hirta*	Wounds	2	2
*Datura metel* L. (Solanaceae)	Almoukaikaii (d)	Leaves	Powder	Drinking		Pain	2	2
*Diospyros mespiliformis* Hochst. Ex (Ebenaceae)	Sunsun(d)	Bark, roots	Decoction	Bathing, drinking		Psychotropic	2	2
*Gardenia erubensis Stapf et* Hutch. (Rubiaceae)	Gulen (d)	Fruit	Decoction	Drinking		Anxiolytic	2	2
*Hymenocardia acida* (Hymenocardiaceae)	Guèrègueniya(d)	Root,	Decoction	Bathing, drinking		Ache	2	2
*Lannea microcarpa* Engl. et K. Krause (Anacardiaceae)	Pekun (d)	Leaves	Powder	Application		Wounds	2	2
*Lannea velutina A. Rich* (Anacardiaceae)	Surugupekun (d)	Bark, leaves	Decoction	Batingh		Ache	2	2
*Leptadenia hastata* (Pers.) Decne. (Asclepiadaceae)	Gnougou (d)	Plant	Decoction	Bathing, drinking		Ache	2	2
*Opilia celtidifolia* (Guill. et Perr) Endl. ex Walp.(Opiliaceae)	Wurunipinba (m)	Leaves, root	Maceration, P	Bathing, application	Salt, *Tamarindus indica*	Ache, wounds	2	4
*Pericopsis laxiflora* (BENTH ex BAK.) van Meeawen (Fabaceae)	Kolokolo (d)	Leaves	Decoction	Bathing, drinking		Ache	2	2
*Pterocarpus erinaceus* Poir (Fabaceae)	Gooni (d)	Gui	Decoction			Ache, anxiolytic	2	4
*Terminalia avicennioides* Guil/. et Perr. (Combretaceae)	Waranpin (dioula)	Leaves	Decoction	Bathing, drinking		Ache, pain	2	2
*Terminalia macroptera* Guill. et Perr. (Combretaceae)	Habè (b)	Root,	Decoction	Wounds		Wounds	2	2
*Waltheria indica L*. (Sterculiaceae)		Plant	Fresh plant, powder	Application	*Parkia biglobosa* fruits	Wounds	2	2
*Xylopia aethiopica* (Dunal) A. Rich. (Annonaceae)	Kanafing (d)	Fruit	Powder	Drinking		Pain	2	2

* F = frequency of citation, ** VU = Value Use. Local names: d = dioula, m = marka, b = bwamu, mo = moore, s = san.

**Table 3:** Results of phytochemical screening of species.

Species	*Annona senegalensis*		*Cassia sieberiana*		*Gardenia sokotensis*		*Ficus thonningii*		*Bauhinia thonningii*		*Securidaga longepedunculata*		
	M	D	M	D	M	D	M	D	M	D	M	D	
**Tannins and polyphenols**	+	+	+	+	+	+	+	+	+	+	+	+	
**Flavonoids**	+	+	+	+	+	+	+	+	+	+	+	+	
**Saponosides**	+	+	+	+	+	+	−	−	+	−	+	+	
**Coumarin**	−	−	+	+	+	+	+	+	−	+	+	+	
**Steroids and triterpenes**	+	+	+	+	+	+	+	+	+	+	+	+	
**Alkaloids**	+	−	+	−	−	+	+	−	−	−	−	+	

Legend: +: Presence of the desired chemical family, −: Absence of the desired chemical family, M = maceration, D = decoction.

## Data Availability

The data and materials used during the current study are available from the corresponding author on reasonable request.

## Supplementary Materials

The following supporting information can be downloaded at: https://www.mdpi.com/article/10.3390/medicines9020010/s1, questionnaire.
