# Supplementary material for: Ethnobotanical and Phytochemical Profiling of Medicinal Plants from Burkina Faso Used to Increase Physical Performance
Source: Medicines (Basel). 2022 Jan 28;9(2):10. doi: 10.3390/medicines9020010 (PMC8878663; doi:10.3390/medicines9020010)
Supplement: Supplementary file 1 [file medicines-09-00010-s001.zip › medicines-1518859-supplementary.pdf]

FICHE D'ENQUETE ETHNOBOTANIQUE

DATE:………………………………ENQUETEUR : ………………………………………

- **Profil de l'informateur**[P1]

✓ Nom prénom …………………………

✓ Profession …………………

✓ Situation matrimoniale…………

✓ Age ……………Sexe : ……………

✓ Ethnie : …………Religion………

✓ Village d'origine ……

✓ Nombre d'années de pratiques : ………………

✓ Niveau d'étude …………………

✓ Spécialiste en traitement de quelle(s) maladie(s) ? (Appellation(s) locale(s), signification, nom(s) français) :……
………

✓ Origine du savoir : ………………………………………………………………………………………
.[P2]

- **Informations sur la zone d'enquête**

✓ Nombre d'habitants : ………………

✓ Ethnie autochtone : ……………

✓ Différentes religions pratiquées :……

✓ Religion majoritaire……………

✓ Langues parlées : …………………

✓ Langue parlée majoritairement ;……………

- **Informations sur les pratiques d'activités physiques et sportives**

✓ Les activités physiques traditionnelles pratiquées dans la localité avec les fréquences /AN (loisirs, culturelles)

……………………………………………… | ………………………………………………
……………………………………………… | ………………………………………………

✓ Les activités sportives pratiquées dans la localité

……………………………………………… | ………………………………………………
……………………………………………… | ………………………………………………
……………………………………………… | ………………………………………………

- **Informations sur les différents troubles et effets rencontrés sur la santé lors de ces pratiques d'activités physiques et sportives**

……………………………………………… | ………………………………………………
……………………………………………… | ………………………………………………
……………………………………………… | ………………………………………………

- **Informations sur les différents modes de préparations des remèdes traditionnels utilisés lors des pratiques d'activités physiques et sportives**

✓ Pour éviter ou soigner les traumatismes mécaniques (entorse, foulure, fractures, plaies, etc.) ……………………………………………………………………………………………………
……………………………………………………………………………………………………

✓ Contre la fatigue ou augmenter l'endurance……………………………………………………
……………………………………………………………………………………………

✓ Pour augmenter sa force musculaire…………………………………………………………
……………………………………………………………………………………………

✓ Accroitre sa force mentale…………………………………………………………………
……………………………………………………………………………………………

✓ Autres motifs ………………………………………………………………………………
……………………………………………………………………………………………………

- **Plantes médicinales utilisées**

:

Nom local ………………………………………………………………
……………………………………………………………………..
Nom français : ……………………………………………………
Binôme latin ………………………………………………………

✓ Effets recherchés ………………………………
………
………
………
………

✓ Parties utilisées ……………………………………………………………………………………………
✓ Stade de développement : …………Mature………Immature………………très Âgée……………
✓ Mode de collecte et instruments utilisés……………………………………………………………
✓ Période de collecte : ……… journée …………………………nuit……Autres… …………
✓ Mode de conditionnement et de conservation avant usage : ………………………………………
✓ Durée de la conservation : ……………………………………………………………………………
✓ Interdits liés à la conservation …………………………………………………………………………

✓ Mode de Préparation
Décoction. ☐ Macération ☐ en poudre ☐ autre forme d'extraction ☐……………
Nature du solvant utilisé : eau ☐ alcool ☐ autre : ☐…………………………………

✓ Mode d'administration
Quantité utilisée …………………………………………………………………………………
Voix d'administration……………………………………………………………………………
Invocation (discours) et/ou geste durant l'administration du remède…………………………
Rituels et sacrifices propitiatoires à la prise du remède : ……………………………………
✓ Durée d'efficacité du remède : ……………………………………………………………………
✓ Interdits

✓ **Plantes associées**

1. Nom local :…………
………………………
Nom en français ……………………………………
Binôme latin…………………………………………
Parties utilisées ……………………………………
Mode d'association…………………………………
……………………………………………………

2. Nom local ……………………………………
………………………………………………
Nom en français ……………………………………
Binôme latin ………………………………………
………………………………………………
Parties utilisées ……… ………………………
Mode d'association…………………………………

✓ **Substances incorporées (origine animale/minérale) :** ……………………………………………
………
………
……………………………………………………………………………………………………

✓ ***Autres informations supplémentaires***
(Effets secondaires liés à l'utilisation du remède)
………
………
………
………
